# Evaluation of the performance of quantitative detection of the *Listeria monocytogenes prfA* locus with droplet digital PCR

**DOI:** 10.1007/s00216-016-9861-9

**Published:** 2016-08-24

**Authors:** Anna Kristina Witte, Susanne Fister, Patrick Mester, Dagmar Schoder, Peter Rossmanith

**Affiliations:** 1 Christian Doppler Laboratory for Monitoring of Microbial Contaminants, Institute of Milk Hygiene, Department for Farm Animals and Veterinary Public Health, University of Veterinary Medicine, Veterinärplatz 1, 1210 Vienna, Austria; 2 Institute of Milk Hygiene, Department for Farm Animals and Veterinary Public Health, University of Veterinary Medicine, Veterinärplatz 1, 1210 Vienna, Austria

**Keywords:** ddPCR, qPCR, *prfA*, *Listeria monocytogenes*, Poisson distribution, Heterogeneous matrix

## Abstract

Fast and reliable pathogen detection is an important issue for human health. Since conventional microbiological methods are rather slow, there is growing interest in detection and quantification using molecular methods. The droplet digital polymerase chain reaction (ddPCR) is a relatively new PCR method for absolute and accurate quantification without external standards. Using the *Listeria monocytogenes* specific *prfA* assay, we focused on the questions of whether the assay was directly transferable to ddPCR and whether ddPCR was suitable for samples derived from heterogeneous matrices, such as foodstuffs that often included inhibitors and a non-target bacterial background flora. Although the *prfA* assay showed suboptimal cluster formation, use of ddPCR for quantification of *L. monocytogenes* from pure bacterial cultures, artificially contaminated cheese, and naturally contaminated foodstuff was satisfactory over a relatively broad dynamic range. Moreover, results demonstrated the outstanding detection limit of one copy. However, while poorer DNA quality, such as resulting from longer storage, can impair ddPCR, *internal amplification control* (IAC) of *prfA* by ddPCR, that is integrated in the genome of *L. monocytogenes* Δ*prfA*, showed even slightly better quantification over a broader dynamic range.

**Electronic supplementary material:**

The online version of this article (doi:10.1007/s00216-016-9861-9) contains supplementary material, which is available to authorized users.

**Graphical Abstract:**
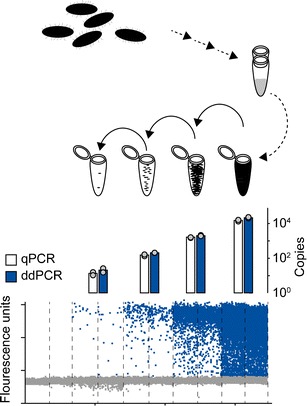
Evaluating the absolute quantification potential of ddPCR targeting *Listeria monocytogenes prfA*

## Introduction

Rapid detection and risk assessment of pathogenic organisms, which can endanger health, are necessary to ensure public interests, such as food safety. Therefore, direct and exact quantification of pathogenic organisms is becoming more and more relevant. This was emphasized, for example, for foodborne pathogens by Hoorfar (2011) [1]. However, microbiological methods based on enrichments do not produce quantitative results necessary for an appropriate risk analysis, and they are also both time- and cost-intensive. Consequently, a major effort is being made to replace these methods with faster and more accurate techniques. These techniques are mostly derived from molecular biology, such as real-time quantitative polymerase chain reaction (qPCR) detection.

qPCR allows for reliable detection and quantification down to one single nucleic acid target per PCR sample, but the down side is that it requires a highly purified template DNA [2]. qPCR quantification directly from complex matrices is rarely performed as these hinder the PCR process itself [1]. This is mostly due to the presence of chemical inhibitors and large numbers of non-target bacterial background flora. Moreover, relevant pathogens are usually present in very low numbers in the environment. Consequently, for example in food diagnostics, a large representative sample (up to 25 g) has to be processed for further analysis. While controls for possible PCR inhibition, such as an *internal amplification control* (IAC) or an *internal sample preparation control* (ISPC), are recommended [3], the possibility of biased quantitative results is still a major concern. The classical qPCR format depends on an external DNA standard that is normally highly pure and thus potentially different in quality from the sample, leading to erroneous results [2].

A possible solution to these inherent problems of pathogen quantification using PCR could be the relatively new PCR format called digital droplet PCR (ddPCR). In this PCR format, the sample is distributed in small droplets (∼20,000), each containing a fraction of the DNA targets of the initial sample. Quantification with ddPCR is performed without an external standard. The underlying algorithm is based on Poisson distribution. According to this distribution, a small number of DNA targets in a large number of droplets lead to the possibility of calculating the overall number of initial DNA target molecules in the sample. Following the PCR run, the samples are screened for droplets, with positive target amplification represented by a fluorescence signal and negative samples lacking such a signal. Subsequently, the initial DNA concentration is calculated from the proportion of negative and positive events and the Poisson distribution prediction [4].

ddPCR has already been tested in a variety of applications. Besides research on cancer and other diseases, ddPCR was inter alia applied to food that contained DNA from genetically modified organisms [5, 6], bacterial pathogens in water samples [7], and for the detection of methicillin-resistant *Staphylococcus aureus* [8].

ddPCR is more attractive than qPCR in diagnostic applications for the following reasons: ddPCR determines the absolute target copy number without the need of an external standard and thus this technique is not limited by the possibilities of DNA standard degradation [9] or DNA extracted from different matrices [10]. Consequently, results from different runs and laboratories show better comparability, which has been demonstrated by Fu et al. (2015) [6]. Additionally, quantification with ddPCR is theoretically less dependent on inhibitors influencing the amplification efficiency than qPCR since it is an end-point measurement [11, 12]. Finally, ddPCR might reduce the possibility of handling errors due to automated generation of the droplets, therefore preventing cross contaminations.

The smaller dynamic range of the ddPCR format (5 log_10_, [4, 13]) compared to qPCR is not a drawback for diagnostic applications, as the target organisms normally occur only in low numbers that are correctly quantified with ddPCR. However, there are still concerns about the possibility of directly transferring already well-established qPCR assays into new ddPCR applications. This is especially relevant for diagnostic purposes, as numerous qPCR assays are established in this field. It would be useful to transfer these assays directly to the ddPCR application.

In this study, we investigated this question illustrated by a well-established qPCR assay amplifying a 274-base pairs (bp) fragment of the *Listeria monocytogenes prfA* locus. The Gram-positive bacterium *L. monocytogenes* is one of the most important foodborne pathogens. Its ubiquitous occurrence in the environment combined with its ability to multiply at refrigeration temperatures has led to many food-related outbreaks in the past with often fatal consequences [14]. In addition to its general importance in the food sector, *L. monocytogenes* was also chosen as a model organism as there is much experience with the *prfA* assay. This assay, specific for *L. monocytogenes* [15], has already been thoroughly evaluated and it has been demonstrated that it amplifies one single DNA target molecule. In addition, a Δ*prfA* ISPC [16] is available for this assay. It comprises a Δ*prfA L. monocytogenes* strain including an artificial single-copy IAC sequence of 100 bp. An ISPC serves as an additional control comparable to the IAC, but covers the complete analytical chain, including sample preparation and DNA isolation/purification as well as PCR amplification [2]. It is performed in a duplex format using identical primers and a different labeled probe.

**Fig. 1:**
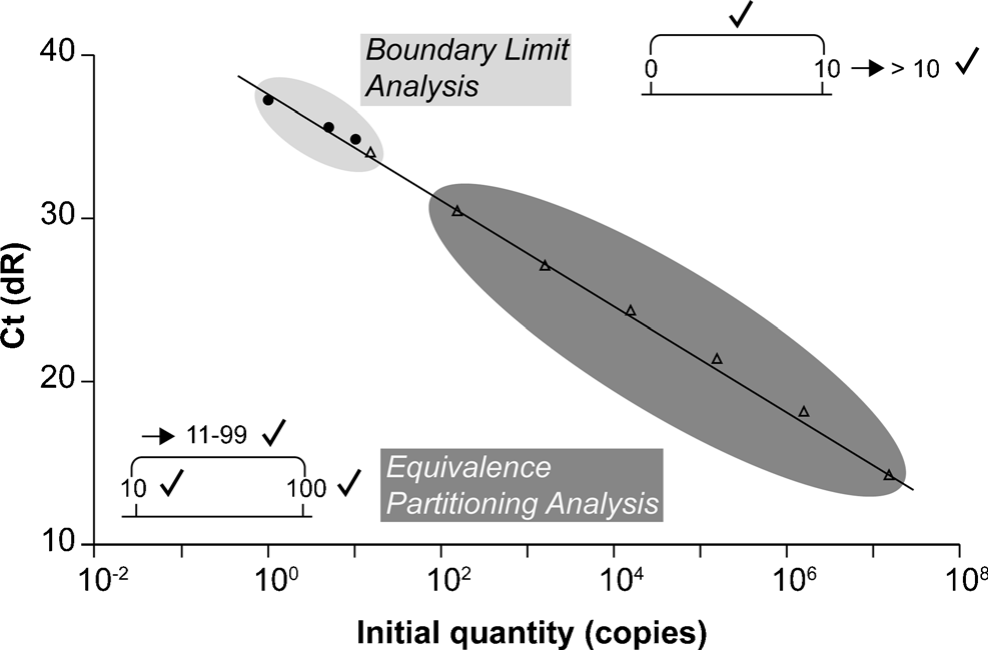
Testing methods of *Boundary Limit Analysis* and *Equivalence Partitioning Analysis* (modified after Rossmanith and Wagner (2011) [17]). The plot demonstrates the ranges of *Boundary Limit Analysis* and conventional *Equivalence Partitioning Analysis* using the example of the calibration curve of a qPCR assay. *Equivalence Partitioning Analysis* covers the range of >10^1^ to 10^7^ initial DNA template copies. *Boundary Limit Analysis* can be applied for the range <10^1^ initial DNA template copies

In addition to the issue of direct transfer of the qPCR assay to the ddPCR format, *prfA* assay performance was evaluated in the ddPCR format. On one hand, this was performed at the DNA level using *Equivalence Partitioning Analysis* based on calibration curves. This analysis is based on verification of representative values in this specific range and the assumption that accuracy of the system is provided for all intermediate values within this range. On the other hand, ddPCR was tested with the *Boundary Limit Analysis* which tests the limiting range using Poisson distribution and assumes that the results of other values are accurate when the assay performs correctly in the limiting range as demonstrated by Rossmanith and Wagner (2011) [17] (Fig. 1). The in vivo application was evaluated by analyzing artificially contaminated specimens and naturally contaminated acid curd cheese and alpine hard cheese.

## Materials and methods

### DNA isolation

DNA was isolated using the NucleoSpin tissue kit (Macherey Nagel) following protocol instructions for Gram-positive bacteria. The DNA was eluted twice with 50 μl ddH_2_O (70 °C).

### DNA standard for real-time PCR quantification

One milliliter of a *L. monocytogenes* (strain EGDe) overnight culture was used for DNA isolation. The DNA concentration was measured with the Qubit ds Broad Range Kit (Invitrogen). The copy number of the single-copy *prfA* gene was calculated using the molecular weight (1 ng of DNA equals 3.1 × 10^5^ copies of the genome).

### qPCR

One qPCR reaction of 25 μl final volume contained 2.5 μl 10× reaction buffer (Invitrogen), 3.5 mM MgCl_2_, 12.5 pmol of each primer, 6.25 pmol of each probe, 5 nmol each dATP, dTTP, dGPT, and dCTP, 1.5 U of Platinum Taq (Invitrogen, Lofer, Austria), and 5 μl of template DNA. The “traditional” *prfA* qPCR was performed as previously published in an Mx3000p real-time PCR thermocycler (Stratagene) with initial denaturation at 94 °C for 2 min, amplification in 45 cycles at 94 °C for 15 s and 64 °C for 1 min [18]. All qPCRs were performed in duplicate. In addition to the “traditional” *prfA* qPCR, a second program was created in accordance with parameters recommended by the ddPCR supplier (Bio-Rad). To cope with the chemicals, the ramp time had to be reduced to 2 °C/s; therefore, a program was created that holds every second degree as a one second step. The data were analyzed with the MxPro software.

### ddPCR

One ddPCR reaction contained 10 μl of ddPCR Master Mix for Probes (Bio-Rad, Munich, Germany), 12.5 pmol of each primer, 6.25 pmol of each probe, and 5 μl of template DNA. Samples were prepared in duplicate with 10 % additional volume and droplets generated (QX100 droplet generator, Bio-Rad, Munich, Germany). PCR was performed as following: initial denaturation at 95 °C for 10 min, amplification in 40 cycles at 95 °C for 30 s and 60 °C for 1 min, and enzyme deactivation at 98 °C for 10 min. For all steps, a ramp time of 2 °C/s was used. Afterwards, the droplets were analyzed in the QX100 droplet reader (Bio-Rad, Munich, Germany). The data were analyzed with the Quantasoft software 1.7 (Bio-Rad, Munich, Germany).

### Poisson distribution-based approach

To receive one, three, and ten copies, the DNA of the lowest log-scale standard “15” was diluted (1: 15, 1:5, 1:1.5) and used as template DNA for 30 (one and three copies) and 20 (ten copies) PCRs. Data of the qPCR were rounded mathematically. Unlike other experiments with the ddPCR, the absolute number of positive droplets per sample was used instead of the “copies/20 μl well” (corresponds to copies in one reaction): The Quantasoft software estimates the “copies/20 μl well” based on the number of analyzed droplets in relation to the expected 20,000 droplets. In ddPCR, before amplification, the sample is divided in 20,000 reactions (droplets) that either contain DNA or not (distribution of DNA is based on Poisson distribution) and after PCR these droplets are either positive (contain DNA) or not (contain no DNA). The Quantasoft software calculates the initial DNA copies using the information of the number of positive and negative droplets and the number of analyzed droplets (due to technical reasons not all 20,000 droplets were analyzed) by means of Poisson statistics.

When working with low copy numbers (<10 copies), DNA is distributed according to Poisson statistics which means that a relative high number of negative samples is expected. In the case of working with one copy per sample, statistically 37 % of all samples are expected to be negative. Furthermore, statistically 37 % of the samples are expected to have one copy, 18 % two copies, and so on. Since approximately only 15,000 out of 20,000 droplets in samples were analyzed, 25 % more negative samples were expected in ddPCR compared to qPCR. Samples containing one copy or more were corrected by the Quantasoft software depending on the number of analyzed droplets (e.g., when detecting 1 positive droplet and 16,003 droplets were analyzed, the estimated “copies/20 μl well” are 1.4.). Thus, to simplify matters and to take the expected higher number of negative samples into account, the absolute number of positive droplets was used and the final result compared to the qPCR data whereby a deviation of 25 % was expected. The data shown in the results section represent the distribution of one of two independent experiments. When fitting data to a Poisson distribution, no other statistics are applied [19].

### Bacterial strains and culture conditions

*L. monocytogenes* EGDe (1/2a, internal number 2964) as well as *ΔprfA L. monocytogenes* EGDe (1/2a) were part of the collection of bacterial strains at the Institute of Milk Hygiene, Milk Technology and Food Science, University of Veterinary Medicine, Vienna, Austria. All bacterial strains were grown overnight in tryptone soya broth with 0.6 % (*w*/*v*) yeast extract (TSB-Y; Oxoid, Hampshire, UK) at 37 °C. Enumeration of bacterial suspensions was performed using the plate count method.

### Artificially contaminated food samples

Gouda cheese was purchased in a local supermarket. A 3-h culture of *L. monocytogenes* EGDe and Δ*prfA* was centrifuged for 5 min at 8000×*g* and washed in 1× PBS. The cultures were adjusted to an optical density OD_600_ = 0.6 in PBS, assuming that a culture with OD_600_ = 0.6 contains 10^8^ CFU/ml. A 10-fold dilution series in PBS was prepared and 100 and 400 μl of the relevant dilution (10^6^ to 10^2^ CFU/ml) added to the 6.25 g (matrix lysis) and 25 g (combined enrichment/qPCR and ISO-11290 [20, 21]) homogenized cheese, respectively. Samples were prepared in four repetitions. The number of CFU was obtained by plating the cells onto tryptone soya agar with 0.6 % yeast (Oxoid).

Matrix lysis was performed as previously described [22]: 10 ml matrix lysis buffer (50 mM Tris pH 7.6, 1 M MgCl_2_) was added to 6.25 g cheese and homogenized twice in the Stomacher 400 (Seward, London, UK) for 2 min. The cheese was transferred to 50 ml tubes and made up with lysis buffer to a volume of 45 ml. Samples were incubated at 37 °C for 30 min, shaking horizontally at 200 rpm in a water bath, and afterwards centrifuged for 30 min at 3220×*g* at 30 °C. The pellet was resuspended in 45 ml wash buffer (1× PBS, 0.35 % Lutensol AO-07) and the samples were again incubated at 37 °C for 30 min, shaking horizontally at 200 rpm in a water bath. The remaining pellet containing the bacteria, was collected by centrifugation for 30 min at 3220×*g* at 30 °C, and transferred to a 2-ml tube (Eppendorf, Hamburg, Germany) following one washing step in 1.5 ml 1× PBS (8000×*g*, 5 min). The complete pellet was used for DNA isolation.

### Combined enrichment/qPCR method

The samples were grown and collected as described previously [18, 23]: 25 g of cheese was incubated in 225 ml half-Fraser medium (Oxoid), according to ISO-11290-1. After 24 h at 30 °C, a 9-ml aliquot was centrifuged for 2 min at 50 g and afterwards the supernatant was centrifuged at 3220×*g* for 10 min. The complete bacterial pellet was used for DNA isolation.

Primers and probes:

**Table:** 

Name	Sequence	
LIP1	5′-GAT ACA GAA ACA TCG GTT GGC-3′	(Eurofins, Ebersberg, Germany)
LIP2	5′-GTG TAA TCT TGA TGC CAT CAG G-3′	(Eurofins, Ebersberg, Germany)
LIP probe2	5′-FAM-CAG GAT TAA AAG TTG ACC GCA-MGB-3′	(Fisher Scientific, Vienna, Austria)
p-lucLm 5	5′-HEX-TTC GAA ATG TCC GTT CGG TTG GC-BHQ1-3′	(Eurofins, Ebersberg, Germany)

## Results and discussion

### Performance of *prfA* and ∆*prfA* assays in the ddPCR

Evaluating the performance of a qPCR assay with a log-scale standard curve is conventionally made over the range of six log_10_ units with a lower limit of approximately 25 DNA copies. This is performed using the *Comparative Threshold* method (Ct-method), which is equivalent to *Equivalence Partitioning*, a widespread test system. Implicit is the assumption that if test results for a few representative experimental values are correct (e.g. 100 and 1000 copies) then the system also provides accuracy over the range of all intermediate values (e.g. 101 to 999 copies; Fig. 1).

An alternative testing method is *Boundary Limit Analysis*. This operates over the range 1 to 10 copies of initial DNA template for qPCR (Fig. 1). It assumes that if a system operates well over the limiting range (e.g. 1 to 10 copies), it will also work well for all other ranges (e.g. >10 copies). According to *Boundary Limit Analysis*, one practical approach to testing is comparison of the characteristic pattern of Poisson distribution with the actual results obtained [17, 19]. As outlined in the “Introduction” section, the *prfA* assay was evaluated using *Boundary Limit Analysis*. Therefore, it was obvious also to test the application in ddPCR with this algorithm as *Boundary Limit Analysis* is most accurate in terms of the qualitative and quantitative resolution of the method.

#### Adjustment of *prfA* and ∆*prfA* to special requirements of ddPCR

**Fig. 2:**
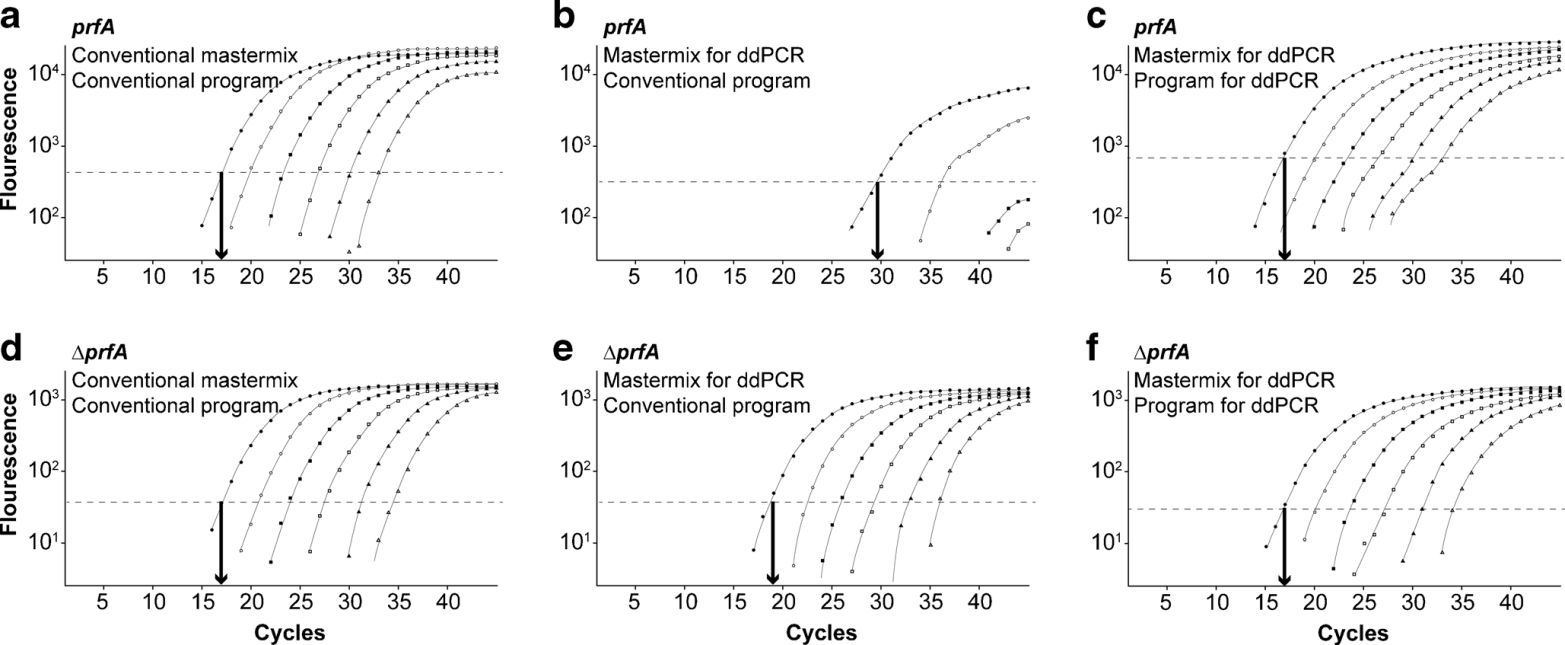
qPCR of *prfA* and the internal sample process control Δ*prfA* with different mastermixes and different PCR programs. Various amounts of genomic DNA of *L. monocytogenes* EGDe (**a**–**c**) and Δ*prfA* (**d**–**f**) (1.5 × 10^6^ (*black circles*) to 1.5 × 10^1^ (*white triangles*) copies per sample) were amplified with the conventional mastermix (**a**, **d**), the one for ddPCR (**b**, **c**, **e**, **f**) with the conventional PCR program (**a**, **b**, **d**, **e**) and with the program special developed for ddPCR (**c**, **f**)

Before testing a specific diagnostic qPCR assay in the ddPCR, it is necessary to test if the PCR runs properly under the chemical and physical conditions necessary for ddPCR. It uses a special mastermix adapted to the generation of droplets and a special ramp time necessary for the correct heat transfer into the droplets. Thus, the ddPCR mastermix was tested and qPCR was performed with different genomic *L. monocytogenes* EGDe DNA as well as *L. monocytogenes* ∆*prfA* DNA concentrations (ranging from 1.5 × 10^1^ to 1.5 × 10^6^ copies) and compared to the “traditional” mastermix. Additionally, the impact of the ramp time adapted ddPCR program (2 °C/s ramp time) compared to the original qPCR program was investigated (Fig. 2).

Strongly increased Ct values reflecting reduced amplification efficiency were obtained using the ddPCR mastermix for the *prfA* assay when compared to the standard qPCR mastermix (Fig. 2b). When the ramp time and the temperatures were rendered as recommended by the supplier of the ddPCR, the *prfA* assay worked well (Fig. 2c). Though the amplification curves appeared flatter after adaptation to the ddPCR program, the Ct values were almost identical to the standard qPCR and thus the ddPCR mastermix worked for the *prfA* assay (Fig. 2c). Nevertheless, these preliminary results were obtained using the conventional qPCR cycler and tubes containing 20 μl aqueous solution (but no droplets).

Analogous testing of the ∆*prfA* assay resulted in the same effects on the obtained Ct values, but with a less pronounced shift of three cycles compared to 13 cycles as observed for the *prfA* assay (Fig. 2e, b). After adaptation of the ∆*prfA* assay, the Ct values of both assays matched (Fig. 2c, f).

#### Performance analysis at DNA level

##### Equivalent partitioning

Since it has been shown that *prfA* and ∆*prfA* assays work under the adapted ddPCR protocol, the performances of the assays in the ddPCR were tested on the BioRad platform. This was performed with increasing concentrations of template DNA as a conventional standard curve, as a simplex format and also a duplex format.

**Fig. 3:**
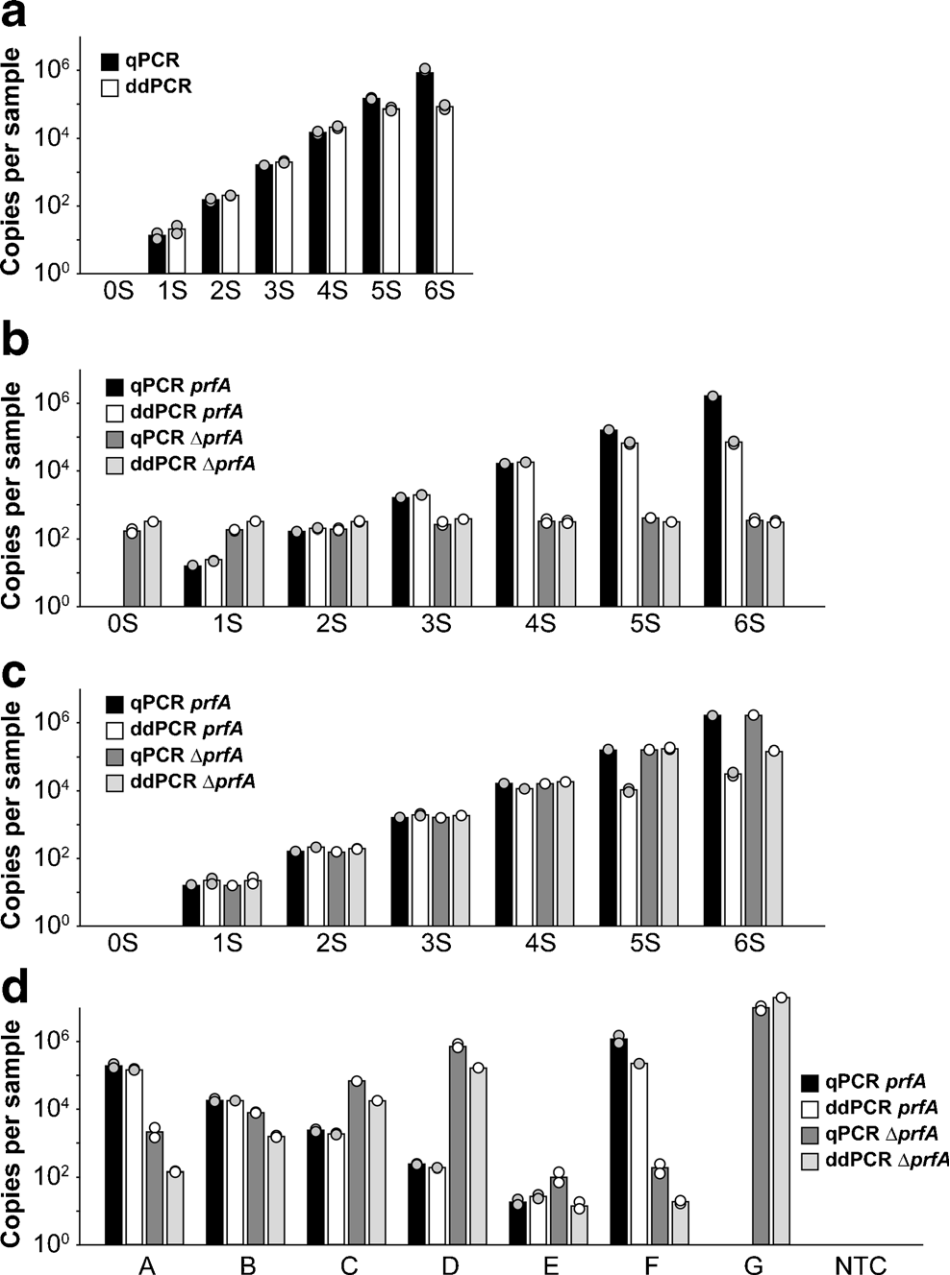
Standard curves with qPCR and ddPCR. Different DNA concentrations from pure cultures of *L. monocytogenes* EGDe and Δ*prfA* were tested with qPCR and ddPCR. **a** Increasing concentrations from 1.5 × 10^1^ to 1.5 × 10^6^ copies per sample of *L. monocytogenes* EGDe. **b** Increasing concentrations of *L. monocytogenes* EGDe (from 1.5 × 10^1^ to 1.5 × 10^6^ copies per sample) with a constant amount of Δ*prfA* (1.5 × 10^2^ copies per sample). **c** Increasing concentrations of *L. monocytogenes* EGDe and Δ*prfA* (1.5 × 10^1^ to 1.5 × 10^6^). **d** Different concentrations of *L. monocytogenes* EGDe and Δ*prfA*: *A*, 1.5 × 10^5^ EGDe and 1.5 × 10^2^ Δ*prfA*; *B*, 1.5 × 10^4^ EGDe and 1.5 × 10^3^ Δ*prfA*; *C*, 1.5 × 10^3^ EGDe and 1.5 × 10^4^ Δ*prfA*; *D*, 1.5 × 10^2^ EGDe and 1.5 × 10^5^ Δ*prfA*; *E*, 1.5 × 10^1^ EGDe and 1.5 × 10^1^ Δ*prfA*; *F*, 1.5 × 10^6^ EGDe and 1.5 × 10^1^ Δ*prfA*; *G*, 1.5 × 10^1^ EGDe and 1.5 × 10^6^ Δ*prfA*; *NTC*, no template control. Duplicates are presented respectively

Overall, there was a high degree of similarity between the results of qPCR and ddPCR when low DNA concentrations were amplified in the simplex reaction using *L. monocytogenes* EGDe DNA. However, at higher concentrations, the quantitative resolution of the ddPCR was not given (Fig. 3a), which is based on the method design using approximately 20,000 droplets per sample. This restricted number of droplets is part of the design of the ddPCR method [4, 13]. Quantification over this range of concentrations is sufficient for most applications of this method. Especially in respect to the investigation of food samples, the high concentration range is neither interesting nor crucial since food pathogens are usually present in very low numbers in the environment. Moreover, low legal detection limits such as 100 CFU/g for *L. monocytogenes* necessitate good quantitative performance over the dynamic range given by ddPCR.

In the duplex format, these results were confirmed with *L. monocytogenes* EGDe DNA combined with a constant amount of *L. monocytogenes* ∆*prfA* DNA (Fig. 3b), and combined with equal and opposed concentrations of *L. monocytogenes* ∆*prfA* DNA (Fig. 3c, d). At low concentrations, quantification of the ddPCR is equal to that of qPCR. The reaction of the other target was only influenced when very high DNA concentrations of one target were applied (Fig. 3d, concentration C).

##### Boundary limit analysis

The detection limit was tested as demonstrated for the *prfA* assay in the qPCR to validate the *prfA* assay in the ddPCR per se [19]. The rationale was that the ddPCR is an enzymatic assay and thus, per se validation as usually applied in analytical and organic chemistry [24] should be performed and not only a comparison to other methods.

**Fig. 4:**
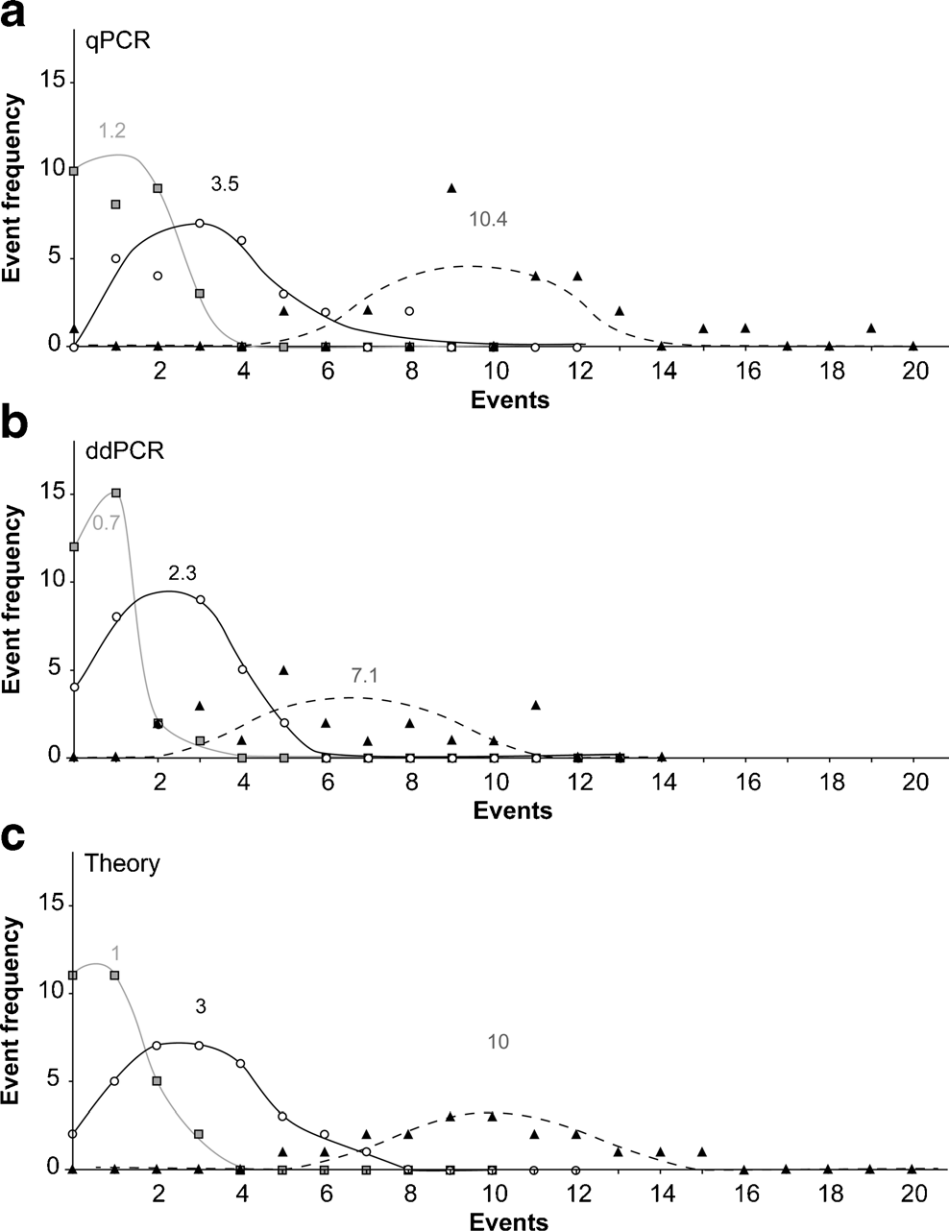
Poisson analysis. One, three, and ten copies of *L. monocytogenes prfA* were amplified in qPCR (**a**) and ddPCR (**b**). By plotting the events against event frequency, the curves approach the Poisson distribution pattern. The curves of **c** show the theoretical distribution

As outlined above, *Boundary Limit Analysis* using the Poisson distribution is an appropriate test. In comparison to the conventional standard-based *Equivalent Partitioning* method, *Boundary Limit Analysis* gives clear evidence of the performance of a method in the boundary limit region. In the case of qPCR and ddPCR, this can be theoretically shown down to the limit of one target molecule. Therefore, one, three, and ten copy numbers were amplified using ddPCR. Within this copy number range, the DNA distributes according to the Poisson distribution [19, 25]. Thirty replicates for one and three copies and 20 replicates for ten copies were quantified with *prfA* ddPCR. The focus of analysis was whether total copy number and the average of each batch (one, three, and ten) matches with the expected copy number and whether the results of the batches are correctly connected to each other. In addition, it was questioned whether the pattern of positive and negative samples and the distribution of the actual initial copy numbers of the discrete samples correlate with the Poisson distribution. The average values in the qPCR control experiment for the batches including one, three, and ten copies are 1.2, 3.5, and 10.4 copies, respectively. This matches the expected values (Fig. 4a). This was also true for the positive and negative distribution of the samples.

Analysis of the number of positive droplets of the analogous experiment performed using the ddPCR platform (Fig. 4b) resulted in 0.7, 2.3, and 7.1 copies for the batches including theoretically one, three, and ten copies. The analysis was performed based on the actual number of positive droplets and not on the algorithm of the QuantaSoft software called “copies/20 μl well” (see also “Material and methods”). This was done since about 25 % of the droplets are not analyzed with ddPCR for mechanical reasons. Therefore the expected values of ddPCR are only 75 % of those from qPCR. Accordingly, the results obtained by ddPCR (0.7, 2.3, and 7.1 copies) match the results obtained by qPCR (Fig. 4). Additionally, the obtained values of 0.7, 2.3, and 7.1 copies for the batches correlate adequately with each other. The number of negative events in the batch for one copy in ddPCR also fitted with 12 from 30 in total (in the qPCR 10 of 30).

In summary, it has been demonstrated in this study that quantitative as well as qualitative detection limits of the *prfA* assay in ddPCR is one copy number and thus it is as sensitive as *prfA* qPCR. A similar outstanding detection limit is suggested by the detection of HIV DNA with ddPCR when increasing the number of replicates [26].

#### Droplet cluster

**Fig. 5:**
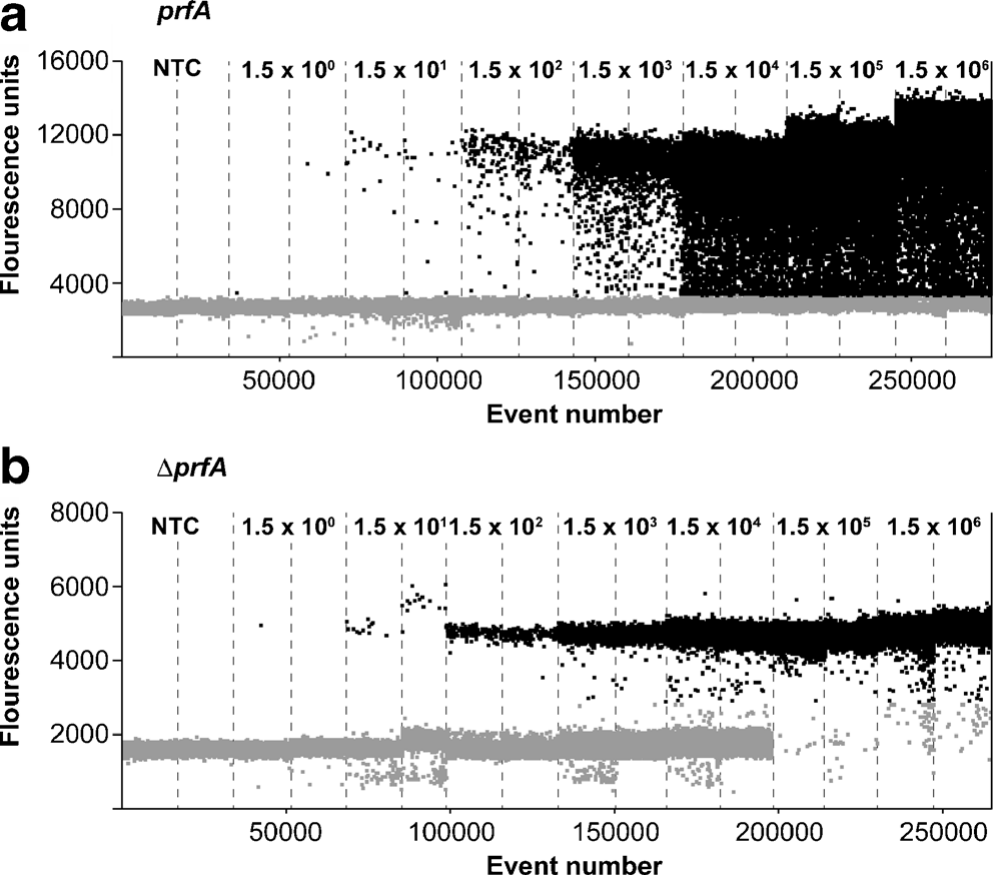
Droplet clustering in the ddPCR of *prfA* (*L. monocytogenes* EGDe) and Δ*prfA* (*L. monocytogenes* Δ*prfA*). Droplet formation with *prfA* and Δ*prfA* is demonstrated with increasing concentrations from 1.5 × 10^1^ to 1.5 × 10^6^ copies per sample of *L. monocytogenes* EGDe (**a**) and Δ*prfA* (**b**)

Despite excellent quantification resolution of the *prfA* and ∆*prfA* assays in ddPCR, one phenomenon was noticeable. Positive droplets of the *prfA* assay were not as clearly separated from the negative droplets as those of the ∆*prfA* assay, which is based on lower fluorescence measurements from these droplets (Fig. 5). In the 1D plot of the analysis software, this is visualized as droplet “rain” (Fig. 5a) as, for example, similarly found with ddPCR for the detection of *Ralstonia solanacearum* [27]. This phenomenon makes it somewhat difficult to set a definite threshold. Thus, single droplets may appear positive in ddPCR in samples where no signal is detected in qPCR. This was demonstrated by pipetting eight times the no template control (NTC) in ddPCR for confirmation (see Electronic Supplementary Material (ESM) Fig. S1). In this control experiment, the single droplets with intermediate fluorescence are most improbably positive, but nevertheless indistinguishable from positive droplets appearing in the “rain” of a normal run. These false positive droplets were also found in other studies, where the source remained unclear [26, 28].

The relevance of this effect on qualitative evaluation of samples in the practical application of ddPCR must be interpreted in the following manner. The number of droplets within the “rain” region of the 1D plot comprises approximately 10 % of the total number of positive droplets (Fig. 5a). Therefore this phenomenon is relevant only for the range <10 BCE (bacterial cell equivalents) per sample, were Poisson distribution of the DNA occurs. If we consider that the appearance of droplets in the “rain” section is associated with a probability of 10 %, we can conclude that the appearance of two positive droplets in the “rain” section of the plot for a particular sample with two positive droplets is thereby very unlikely. Since one droplet in this case can be clearly evaluated as positive, the sample can be conclusively classified as positive. Therefore only samples demonstrating one positive droplet in the “rain” region of the 1D plot are prone to be falsely evaluated. Nevertheless, as stated, only 10 % of the positive droplets are associated with these lower fluorescence measurements. Additionally, the likelihood of false positive droplets appearing, which are undistinguishable from the 10 % positive droplets in the rain region, is also rare.

Furthermore, artifacts with high fluorescence in both HEX and FAM channels were identified in the above experiment (ESM Fig. S1) which were similarly noticed by Kiselinova et al. [28]. However, these artifacts can easily be determined as the dots on the plot appear in the HEX and the FAM channel coincidently with aberrant high fluorescence values. This positive signal in both channels is also observed in simplex assays using FAM labeled probes with no HEX dye at all. Therefore the artifacts are easy to detect and these droplets were judged negative.

### Qualitative and quantitative detection of *L. monocytogenes* from artificially contaminated gouda cheese

As presented in the previous section, performance and quantification of *prfA* by ddPCR functioned well with DNA derived from pure bacterial cultures. However, due to heterogeneity, background flora, and inhibitors, quantification using DNA derived from foodstuffs is much more challenging and thus results of in vitro experiments cannot be transferred directly. To investigate the general applicability and performance of ddPCR for qualitative and quantitative detection of *L. monocytogenes* from foodstuffs, gouda cheese samples were artificially contaminated, DNA was extracted and purified following matrix lysis and quantified [22]. Additionally, a control run was performed following 24 h of enrichment [18, 23]. Genomic DNA of *L. monocytogenes* was subsequently quantified using qPCR and ddPCR and the data were additionally compared with the results of ISO11290-1 and ISO11290-2 (ESM Table S1). Two approaches were implemented: (i) Duplex reactions containing both *L. monocytogenes* EGDe at increasing log concentrations and *L. monocytogenes* ∆*prfA* as ISPC at a constant concentration of 1.6 × 10^3^ CFU, and (ii) duplex reactions containing *L. monocytogenes* EGDe at increasing log concentrations combined with *L. monocytogenes* ∆*prfA* at decreasing log concentrations (ESM Fig. S2, Table S1). To cover a broad concentration range, the bacteria were tested at concentrations from approximately 20 bacteria per sample (3.2 CFU/g) up to 2 × 10^5^ bacteria per sample (3.2 × 10^5^ CFU/g). ISO 11290-1 and ISO 11290-2 were performed in simplex reactions including *L. monocytogenes* EGDe at increasing log concentrations.

The qualitative ISO 11290-1 and both PCR methods following enrichment were positive at all concentrations. However, due to enrichment, the results are not quantitative and the values for all PCR samples were accordingly very high. The obtained values from both PCR methods here differ more compared with other experiments: qPCR results are higher than those of ddPCR (ESM Fig. S3). This result was not surprising. Due to the limited number of droplets (20,000), ddPCR is restricted in its dynamic range and thus quantification of more than ∼10^5^ copies per 20 μl reaction is not possible [4, 13].

**Fig. 6:**
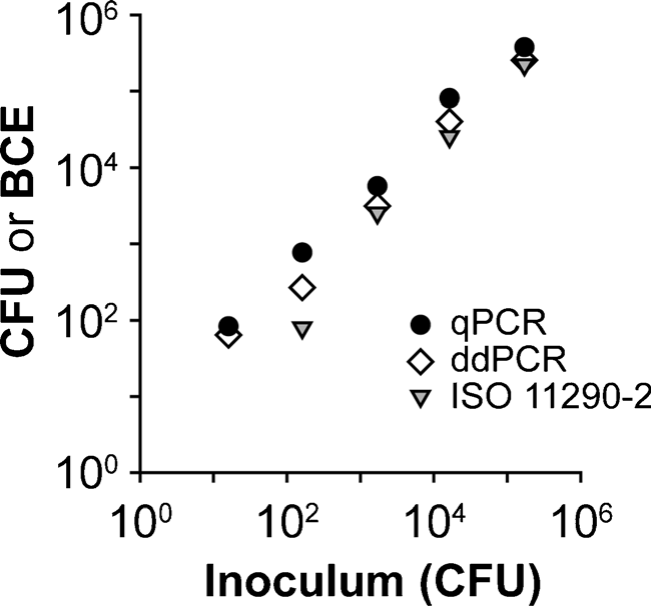
qPCR, ddPCR, and quantitative ISO 11290-2 from artificially contaminated cheese samples. Both qPCR and ddPCR can quantify *L. monocytogenes* over a broad range of concentrations from the cheese samples. Furthermore, both PCR methods correlate well with quantitative ISO 11290-2. However, it is not as sensitive as the PCR methods. Qualitative ISO 11290-1 was positive for all samples. The averages of two independent experiments (each of two samples in duplicates) are demonstrated. Colony forming units (CFU) and bacterial cell equivalents (BCE) are indicated for the 6.25-g sample tested

The results of ddPCR after matrix lysis correlate well with quantitative ISO 11920-2 and qPCR (Fig. 6, ESM Table S1). As expected, quantification with the PCR methods was more sensitive than quantification with ISO 11290-2 that did not detect *L. monocytogenes* at the lowest concentrations. *L. monocytogenes* ∆*prfA* quantification also correlated well with the inoculum (ESM Fig. S2, Table S1) in ddPCR.

There was no suggestion that ddPCR is influenced by background flora or related phenomena in the food samples. Despite the mentioned suboptimal droplet clustering of the *prfA* assay, and thus the ambiguous threshold, quantification using ddPCR in practice was conclusive. However, it must be stated that changes in the manually defined threshold affect quantification of the *prfA* assay by about 10 %. Nevertheless, this deviation is comparably low and sufficient for diagnostic applications. As discussed by other researchers, the threshold issue is a weak point for ddPCR, in particular for comparing inter-laboratory results associated with low DNA concentrations [29]. Thus, one approach to reduce the deviation is the development of software to improve the threshold setting [27, 30].

### Quantification of *L. monocytogenes* from naturally contaminated specimens

After demonstrating the applicability of ddPCR for quantification of bacteria from artificially contaminated food, we compared its performance against qPCR in respect of naturally contaminated specimens. A total of 13 foodstuff batches from two different food classes (acid cured cheese and alpine cheese) were investigated. These included samples from the acid curd cheese-related *L. monocytogenes* outbreak in Austria and Germany from 2010 [14]. Due to the relative rarity of naturally contaminated *L. monocytogenes* samples, half of the samples were old DNA samples from previous DNA extractions stored at −20 °C, while the other half were freshly extracted from naturally contaminated alpine cheese using matrix lysis. All data from qPCR, ddPCR, as well the respective ISO 11290-1 and ISO 11290-2 results are summarized in Table 1.

**Table 1:** ISO 11290-1 and ISO 11290-2, qPCR, and ddPCR with naturally contaminated food samples

				Positive samples/total samples		Quantification (BCE/g)		Validated as positive (+)/negative (−)		
	Batch	ISO 11290-2 (CFU/g)	ISO 11290-1	qPCR	ddPCR	qPCR	ddPCR	ISO	qPCR	ddPCR
Alpine cheese (2014)	I	n.p.	4/5	16/16	24/24	3.6 × 10^2^	2.7 × 10^2^	+	+	+
	II	n.p.	4/5	18/20	17/18	1.2 × 10^3^	1.7 × 10^2^	+	+	+
	III	n.p.	3/5	20/20	22/22	3.3 × 10^2^	2.5 × 10^2^	+	+	+
	IV	n.p.	4/5	18/20	14/20	1.5 × 10^2^	1.5 × 10^1^	+	+	+
	V	n.p.	5/5	13/16	10/16	2.6 × 10^2^	8.6 × 10^1^	+	+	+
*Quargel* cheese (2010)	2	1.1 × 10^7^	5/5	8/8	8/8	8.7 × 10^5^	2.3 × 10^5^	+	+	+
	3	2.2 × 10^4^	5/5	8/8	8/8	1.1 × 10^4^	2.3 × 10^3^	+	+	+
	4	1.6 × 10^4^	5/5	8/8	8/8	3.7 × 10^3^	7.6 × 10^2^	+	+	+
	5	1.2 × 10^6^	5/5	8/8	8/8	1.7 × 10^5^	4.3 × 10^3^	+	+	+
	6	2.8 × 10^3^	5/5	8/8	2/8	1.3 × 10^3^	3.3 × 10^1^	+	+	+
	7	8.8 × 10^3^	5/5	8/8	5/8	2.3 × 10^2^	1.2 × 10^2^	+	+	+
	8	3.4 × 10^3^	5/5	8/8	3/8	2.7 × 10^3^	3.2 × 10^1^	+	+	+
	10	1.5 × 10^2^	5/5	8/8	8/8	2.6 × 10^3^	3.9 × 10^3^	+	+	+

Results of the PCR methods are summarized as mean values

*n.p*. not performed

In summary, evaluations of ddPCR in comparison with qPCR and ISO 11290-1 and ISO 11290-2 matched well, both quantitatively and qualitatively. All investigated food samples were assessed consistently as positive with all three methods, demonstrating good qualitative performance for ddPCR. Quantitative results are discussed in detail in the following section.

#### Alpine cheese samples (fresh DNA)

A quantitative comparison of the results obtained by qPCR and ddPCR using freshly prepared samples of the alpine cheese is shown in Fig. S4 in the ESM. Here, the application of ISPC reveals that the matrix lysis worked well and the quantitative results of both PCR techniques for the *prfA* assay were also in a similar range (between 10^1^ and 10^3^ BCE per gram for both PCR assays). Results indicate that there was no inhibition of ddPCR. However, in some naturally contaminated alpine cheese samples, higher variation and larger deviations were observed between PCR methods compared with experiments using in vitro DNA templates and artificially contaminated samples (see “Performance of *prfA* and Δ*prfA* assays in the ddPCR” and “Qualitative and quantitative detection of *L. monocytogenes* from artificially contaminated gouda cheese” sections). These samples contained very low DNA numbers per PCR reaction (some samples even below ten copy numbers). Therefore, this deviation results from Poisson distribution of the bacterial targets and the resulting negative samples. This distribution of positive and negative samples is the basis of the quantitative calculation and it therefore accordingly influences the quantitative values obtained. That ddPCR detects fewer positive events than qPCR was also found in the study by Hayden et al. [31]. One reason for this might be that only approximately 75 % of droplets were analyzed, meaning that 25 % of the single-copy samples were not detected (see also Poisson validation section).

#### *Quargel* cheese (DNA from storage at −20 °C)

Quantitative results of both PCR methods also deviated when older samples, such as *Quargel* cheese, were compared (ESM Fig. S5a). For example, the positive/negative distribution of results of one *Quargel* cheese sample (number 8) deviated and there were significantly lower quantitative values for ddPCR compared with qPCR (ESM Fig. S5b). Moreover, impaired droplet generation was observed in this sample, which was confirmed by repetitive, independent ddPCR runs (ESM Fig. S5c). Therefore we conclude that the quality of this sample suffered as a result of inhibitory components that may have been present. *Quargel* sample preparation is recognized as particularly challenging matrix. Thus, results from this sample must be critically reviewed and droplet generation parameters should be included in the analysis of suspect samples. One possible explanation could be a high amount of DNA being present in these samples that was shown to affect droplet production [26, 32]. Further, the sample DNA was stored over a longer period at −20 °C, which negatively influences DNA quality [9]. Data obtained directly from fresh samples (performed in 2010) indicate that there was a higher contamination value than shown in this study, which reflects the long-term DNA storage. Nevertheless, the need for long-term DNA storage does not commonly apply to pathogen detection in foodstuffs. Overall, experimental outcomes suggest that DNA quality is even more important for ddPCR than qPCR, although ddPCR is considered more resilient as it is an end-point approach [4, 11].

In conclusion, three important phenomena were observed when naturally contaminated samples were investigated. These account for quantification deviations in using ddPCR compared with qPCR: (i) low contamination levels leading to Poisson distribution of the DNA targets, (ii) sample quality influences droplet generation, and (iii) long-term storage is associated with poor DNA quality. Nevertheless, the quantitative resolution of ddPCR can be assessed as sufficient for practical use and an adequate basis for decision making, especially for fresh specimens.

Interestingly, in contrast to other ddPCR studies [29, 33], ddPCR result deviations from the present study were neither lower nor more precise in respect of quantifying low levels of DNA compared with qPCR.

## Conclusions

As with the study of Morisset et al. [5], the aim of this study was not to reinvestigate ddPCR, but to evaluate its practical applications. We investigated whether the well-established *L. monocytogenes* specific *prfA* qPCR assay is directly transferable to ddPCR and whether ddPCR is suitable for samples derived from heterogeneous matrices that often enclose a non-target bacterial background flora. ddPCR demonstrated excellent quantification of DNA from pure cultures and adequate performance with samples derived from food. However, poorer DNA quality associated with long-term storage impairs ddPCR more severely than qPCR. Despite suboptimal cluster formation, the ddPCR *prfA* assay appears to be suitable for practical applications as only samples where one single droplet appears in the “rain” region cannot clearly be evaluated positive or negative. However, while this scenario is very rare, it is easy to identify these ambiguous samples, which is not possible with other methods. Consequently, conformation of samples yielding a single droplet in the “rain” region necessitates result repetition. Overall, ddPCR was still associated with fewer false results compared with other practical methods, such as the VIDAS system based on antibodies. None the less, we recommend comprehensive adaptations of the qPCR assay to minimize positive and negative droplet overlap and demonstrated that not a trivial matter to transfer qPCR assays to ddPCR.

## Electronic supplementary material

Below is the link to the electronic supplementary material.

ESM 1 (PDF 0.98 mb)

## References

[1] Hoorfar J (2011). Rapid detection, characterization and enumeration of foodborne pathogens. Acta Pathol Microbiol Immunol Scand.

[2] Rossmanith P, Wagner M (2011). The challenge to quantify *Listeria monocytogenes*—a model leading to new aspects in molecular biological food pathogen detection. J Appl Microbiol.

[3] Hoorfar J, Malorny B, Abdulmawjood A, Cook N, Wagner M, Fach P (2004). Practical considerations in design of internal amplification controls for diagnostic PCR assays. J Clin Microbiol.

[4] Hindson BJ, Ness KD, Masquelier DA (2011). High-throughput droplet digital PCR system for absolute quantitation of DNA copy number. Anal Chem.

[5] Morisset D, Štebih D, Milavec M, Gruden K, Žel J (2013). Quantitative analysis of food and feed samples with droplet digital PCR. PLoS One.

[6] Fu W, Zhu P, Wang C, Huang K, Du Z, Tian W (2015). A highly sensitive and specific method for the screening detection of genetically modified organisms based on digital PCR without pretreatment. Sci Rep.

[7] Rothrock MJ, Hiett KL, Kiepper BH, Ingram K, Hinton A (2013). Quantification of zoonotic bacterial pathogens within commercial poultry processing water samples using droplet digital PCR. Adv Microbiol.

[8] Kelley K, Cosman A, Belgrader P, Chapman B, Sullivan DC (2013). Detection of methicillin-resistant *Staphylococcus aureus* by a duplex droplet digital PCR assay. J Clin Microbiol.

[9] Röder B, Frühwirth K, Vogl C, Wagner M, Rossmanith P (2010). Impact of long-term storage on stability of standard DNA for nucleic acid-based methods. J Clin Microbiol.

[10] Burns MJ, Burrell AM, Foy CA (2010). The applicability of digital PCR for the assessment of detection limits in GMO analysis. Eur Food Res Technol.

[11] Hindson CM, Chevillet JR, Briggs HA, Gallichotte EN, Ruf IK, Hindson BJ (2013). Absolute quantification by droplet digital PCR versus analog real-time PCR. Nat Methods.

[12] Dingle TC, Sedlak RH, Cook L, Jerome KR (2013). Tolerance of droplet-digital PCR vs real-time quantitative PCR to inhibitory substances. Clin Chem.

[13] Pinheiro LB, Coleman VA, Hindson CM, Herrmann J, Hindson BJ, Bhat S (2012). Evaluation of a droplet digital polymerase chain reaction format for DNA copy number quantification. Anal Chem.

[14] Fretz R, Sagel U, Ruppitsch W (2010). Listeriosis outbreak caused by acid curd cheese “Quargel”, Austria and Germany 2009. Eurosurveillance.

[15] D’Agostino M, Wagner M, Vazquez-Boland JA (2004). A validated PCR-based method to detect *Listeria monocytogenes* using raw milk as a food model—towards an international standard. J Food Prot.

[16] Frühwirth K, Fuchs S, Mester P, Wagner M, Rossmanith P (2012). Cloning and characterisation of a Δ-prfA *Listeria monocytogenes* strain containing an artificial single copy genomic internal amplification control (IAC) for use as internal sample process control (ISPC). Food Anal Methods.

[17] Rossmanith P, Wagner M (2011). Aspects of systems theory in the analysis and validation of innovative molecular-biological based food pathogen detection methods. Trends Food Sci Technol.

[18] Rossmanith P, Krassnig M, Wagner M, Hein I (2006). Detection of *Listeria monocytogenes* in food using a combined enrichment/real-time PCR method targeting the prfA gene. Res Microbiol.

[19] Rossmanith P, Wagner M (2011). A novel poisson distribution-based approach for testing boundaries of real-time PCR assays for food pathogen quantification. J Food Prot.

[20] Anonymous (1998) Microbiology of food and animal feeding stuffs—horizontal method for the detection and enumeration of *Listeria monocytogenes*—Part 2: enumeration method. Int Organ Stand Geneva 11290–11292

[21] Anonymous (1996) Microbiology of food and animal feeding stuffs—horizontal method for the detection and enumeration of *Listeria monocytogenes*—Part 1: detection method. International Standard ISO 11290-1. Int. Organ. Stand. Geneva

[22] Mester P, Schoder D, Wagner M, Rossmanith P (2014). Rapid sample preparation for molecular biological food analysis based on magnesium chloride. Food Anal Methods.

[23] Rossmanith P, Mester P, Wagner M, Schoder D (2010). Demonstration of the effective performance of a combined enrichment/real-time PCR method targeting the prfA gene of *Listeria monocytogenes* by testing fresh naturally contaminated acid curd cheese. Lett Appl Microbiol.

[24] Kromidas S, Morkowski J (1999) Validierung in der Analytik

[25] Wang Z, Spadoro J (1998) Determination of target copy number of quantitative standards used in PCR-based diagnostic assays. In: Gene Quantif. Birkhäuser, Boston, pp 31–43

[26] Strain MC, Lada SM, Luong T, Rought SE, Gianella S, Terry VH (2013). Highly precise measurement of HIV DNA by droplet digital PCR. PLoS One.

[27] Dreo T, Pirc M, Ramsak Z, Pavsic J, Milavec M, Zel J (2014). Optimising droplet digital PCR analysis approaches for detection and quantification of bacteria: a case study of fire blight and potato brown rot. Anal Bioanal Chem.

[28] Kiselinova M, Pasternak AO, De Spiegelaere W, Vogelaers D, Berkhout B, Vandekerckhove L (2014). Comparison of droplet digital PCR and seminested real-time PCR for quantification of cell-associated HIV-1 RNA. PLoS One.

[29] Svobodová I, Pazourková E, Hořínek A, Novotná M, Calda P, Korabečná M (2015). Performance of droplet digital PCR in non-invasive fetal RHD genotyping—comparison with a routine real-time PCR based approach. PLoS One.

[30] Trypsteen W, Vynck M, De Neve J, Bonczkowski P, Kiselinova M, Malatinkova E (2015). ddpcRquant: threshold determination for single channel droplet digital PCR experiments. Anal Bioanal Chem.

[31] Hayden RT, Gu Z, Ingersoll J, Abdul-Ali D, Shi L, Pounds S (2013). Comparison of droplet digital PCR to real-time PCR for quantitative detection of cytomegalovirus. J Clin Microbiol.

[32] Weerakoon KG, Gordon CA, Gobert GN, Cai P, McManus DP (2016). Optimisation of a droplet digital PCR assay for the diagnosis of *Schistosoma japonicum* infection: a duplex approach with DNA binding dye chemistry. J Microbiol Methods.

[33] Doi H, Uchii K, Takahara T, Matsuhashi S, Yamanaka H, Minamoto T (2015). Use of droplet digital PCR for estimation of fish abundance and biomass in environmental DNA surveys. PLoS One.

