# Supplementary material for: Evaluation of the performance of quantitative detection of the Listeria monocytogenes prfA locus with droplet digital PCR
Source: Anal Bioanal Chem. 2016 Aug 24;408(27):7583–93. doi: 10.1007/s00216-016-9861-9 (PMC5061835; doi:10.1007/s00216-016-9861-9)
Supplement: Supplementary file 1 — (PDF 0.98 mb) [file 216_2016_9861_MOESM1_ESM.pdf]

Analytical and Bioanalytical Chemistry

Electronic Supplementary Material

Evaluation of the performance of quantitative detection of the *Listeria monocytogenes prfA* locus with droplet digital PCR

Anna Kristina Witte, Susanne Fister, Patrick Mester, Dagmar Schoder, Peter Rossmanith

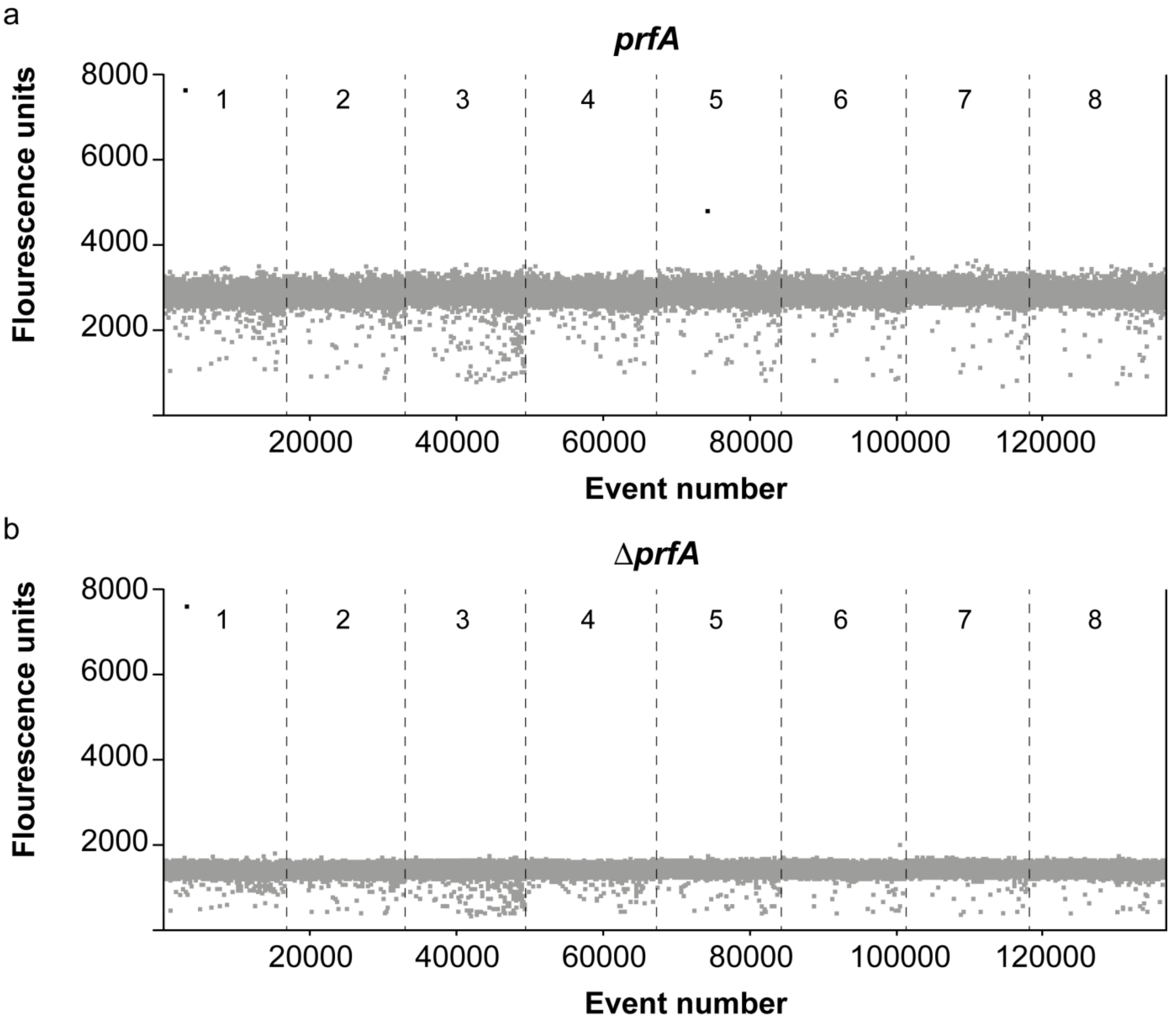

Fig. S1 1D plot if negative controls

1D plot showing negative controls where only mastermix without template was tested eight times in ddPCR. a FAM (*prfA*) and b HEX (Δ*prfA*)

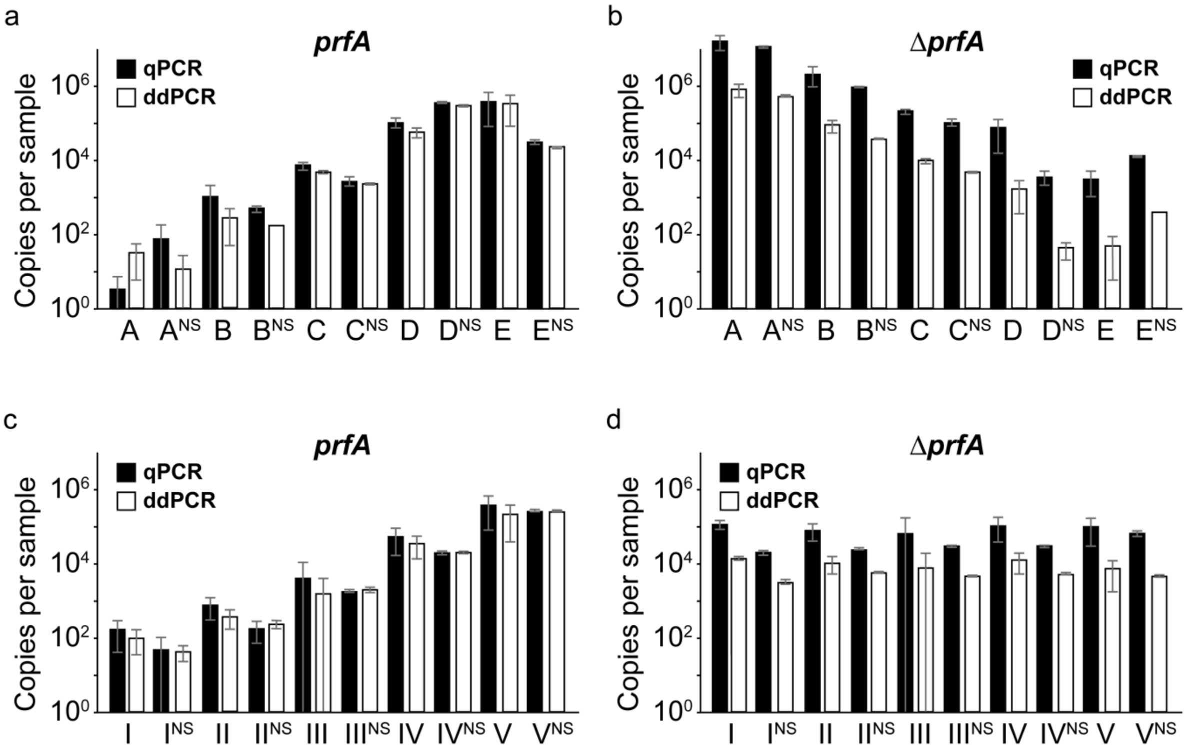

Fig. S2 qPCR and ddPCR of artificially contaminated cheese samples following matrix lysis

qPCR (black) and ddPCR (white) of artificially contaminated samples with opposed amounts of ISPC Δ*prfA* (a, b) and constant ISPC Δ*prfA* (c, d). A/A^{NS} (NS: Nucleospin control): 2 x 10^{1} CFU *L. monocytogenes* EGDe and 2 x 10^{5} CFU Δ*prfA* per sample, B/B^{NS}: 2 x 10^{2} CFU *L. monocytogenes* EGDe and 2 x 10^{4}CFU Δ*prfA* per sample, C/C^{NS}: 2 x 10^{3}CFU *L. monocytogenes* EGDe and 2 x 10^{3} CFU Δ*prfA* per sample, D/D^{NS}: 2 x 10^{4} CFU *L. monocytogenes* EGDe and 2 x 10^{3} CFU Δ*prfA* per sample, E/E^{NS}: 2 x 10^{5}CFU *L. monocytogenes* EGDe and 2 x 10^{1} CFU Δ*prfA* per sample, I/I^{NS} : 2 x 10^{1} CFU *L. monocytogenes* EGDe and 2 x 10^{3} CFU Δ*prfA* per sample, II/II^{NS}: 2 x 10^{2} CFU *L. monocytogenes* EGDe and 2 x 10^{3} CFU Δ*prfA* per sample, III/III^{NS}: 2 x 10^{3} CFU *L. monocytogenes* EGDe and 2 x 10^{3} CFU Δ*prfA* per sample, IV/IV^{NS}: 2 x 10^{4} CFU *L. monocytogenes* EGDe and 2 x 10^{3} CFU Δ*prfA* per sample, V/V^{NS}: 2 x 10^{5} CFU *L. monocytogenes* EGDe and 2 x 10^{3} CFU Δ*prfA* per sample. Bars and error bars represent means and standard deviations of eight PCR results (four times DNA extraction, PCR in duplicate)

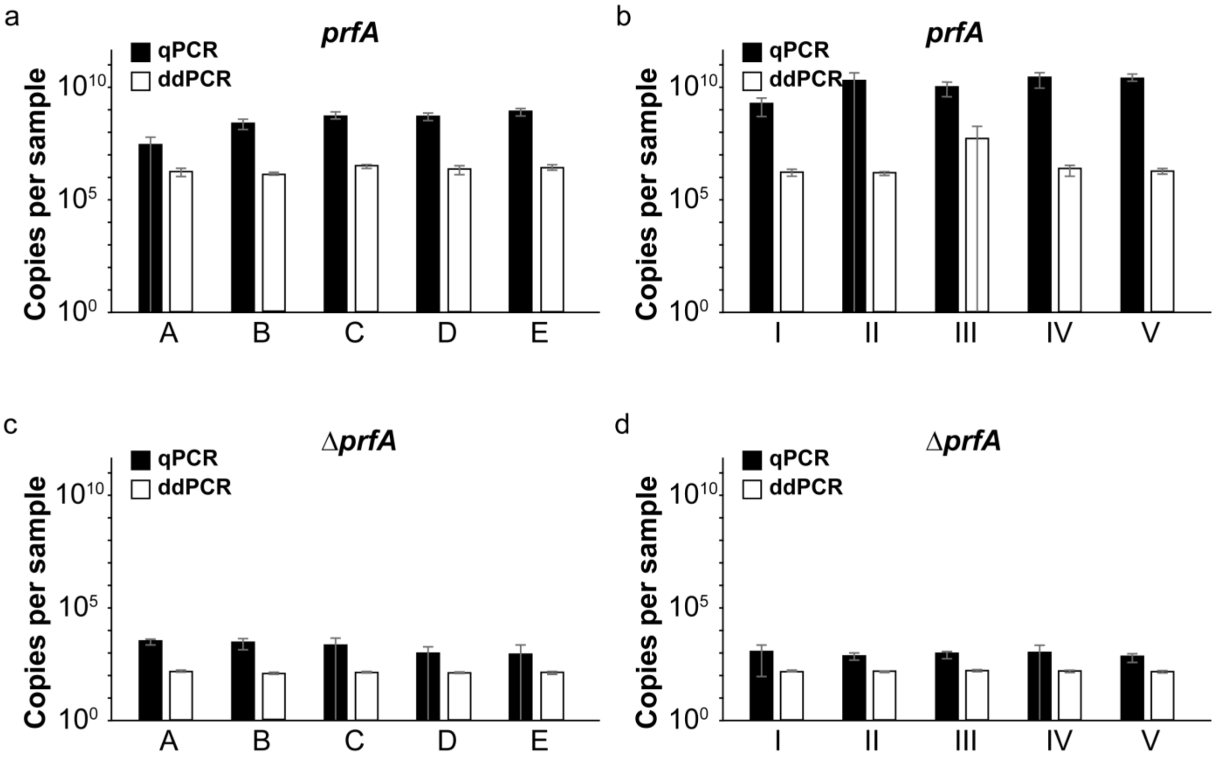

Fig. S3 qPCR and ddPCR of artificially contaminated cheese samples following enrichment

Following 24 hours of enrichment in half Fraser, DNA from artificially contaminated samples was extracted and qPCR (black) and ddPCR (white) performed. A/I: 2 x 10^1 CFU, B/II: 2 x 10^2 CFU*,* C/III: 2 x 10^3 CFU, D/IV:2 x 10^4 CFU, E/V: 2 x 10^5 CFU *L. monocytogenes* EGDe per sample (before enrichment). Δ*prfA* DNA was applied to the mastermix (c and d, ~ 1,600 copies) and detected with the HEX-labelled probe to monitoring the PCR. Columns and error bars represent means and standard deviations of eight PCR results (four times DNA extraction, PCR in duplicate)

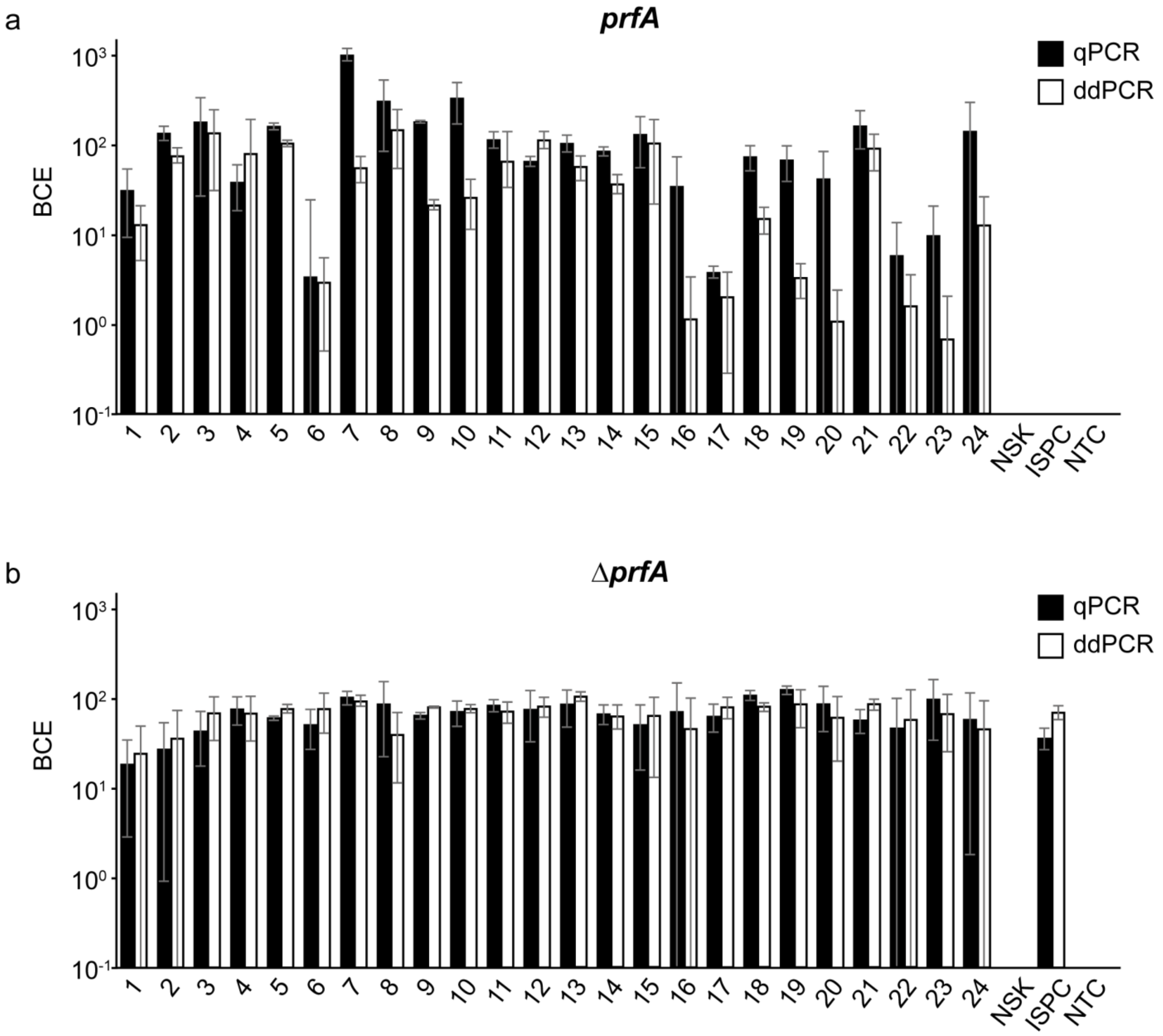

Fig. S4 qPCR and ddPCR of naturally contaminated cheese samples

Naturally contaminated cheese samples were prepared with matrix lysis and the DNA subsequently extracted. **a**. The *prfA* content was determined with qPCR (black) and ddPCR (white). **b**. Before samples were processed, the internal sample process control was added to the cheese and its content determined with qPCR (black) and ddPCR (white). Columns and error bars represent means and standard deviations of four PCR results (preparation and PCR in duplicate)

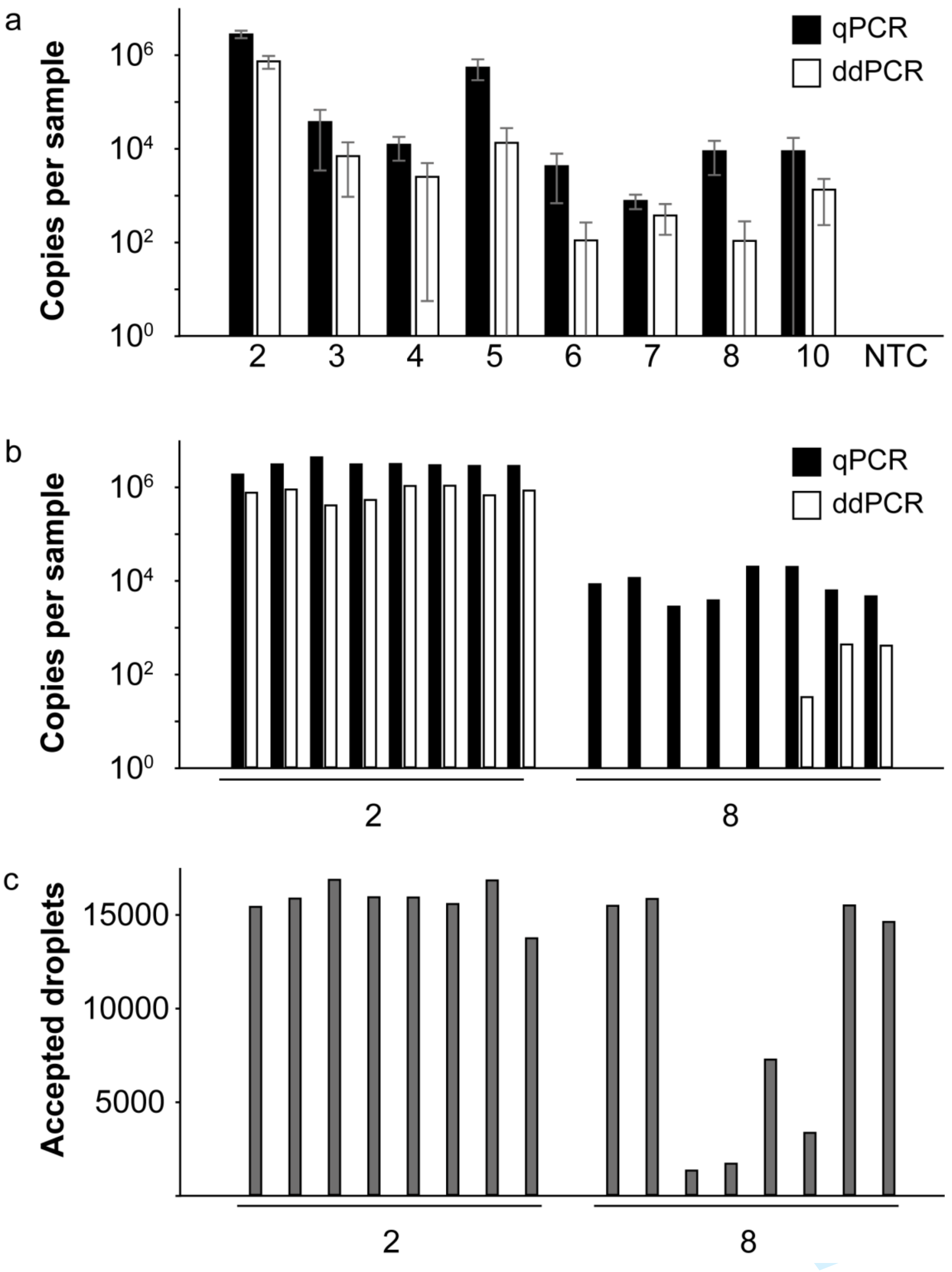

Fig. S5 qPCR and ddPCR of naturally contaminated *Quargel* cheese

a. DNA of naturally contaminated *Quargel* cheese was extracted following sample preparation by matrix lysis and determined using qPCR (black) and ddPCR (white). Columns and error bars represent means and standard deviations from eight replicates of two independent experiments (twice DNA extraction and duplicates of the PCR). **b**. Individual samples of cheeses 2 and 8 in qPCR and ddPCR C. Accepted droplets in ddPCR of samples of cheeses 2 and 8

Table S1 Overview of artificially contaminated cheese samples

	Cheese sample	ISO 11290-1 (qualitative)	ISO 11290-2 (quantitative, CFU)	Inoculum (CFU)	qPCR (BCE)	ddPCR (BCE)	ISPC Inoculum (CFU)	ISPC qPCR (BCE)	ISPC ddPCR (BCE)
**Experiment 1**	A	Positive	Negative	1.5×10^{1}	3.3	3.3×10^{1}	3×10^{5}	1.7×10^{7}	8.4×10^{5}
	B	Positive	Negative	1.5×10^{2}	1.1×10^{3}	2.8×10^{2}	3×10^{4}	2.2×10^{6}	9.1×10^{4}
	C	Positive	2.7×10^{3}	1.5×10^{3}	7.6×10^{3}	5.0×10^{3}	3×10^{3}	2.1×10^{5}	1×10^{4}
	D	Positive	3.1×10^{4}	1.5×10^{4}	1.1×10^{5}	6.0×10^{4}	3×10^{2}	7.6×10^{4}	1.7×10^{3}
	E	Positive	2.6×10^{5}	1.5×10^{5}	4.0×10^{5}	3.5×10^{5}	3×10^{1}	3.4×10^{3}	5.1×10^{1}
**Experiment 2**	I	Positive	Negative	1.8×10^{1}	1.6×10^{2}	9.8×10^{1}	1.6×10^{3}	1×10^{5}	1.3×10^{4}
	II	Positive	7.8×10^{1}	1.8×10^{2}	7.2×10^{2}	3.4×10^{2}	1.6×10^{3}	7.2×10^{4}	9.9×10^{3}
	III	Positive	2×10^{3}	1.8×10^{3}	4.0×10^{3}	1.5×10^{3}	1.6×10^{3}	6.1×10^{4}	7.5×10^{3}
	IV	Positive	1.8×10^{4}	1.8×10^{4}	4.9×10^{4}	3.2×10^{4}	1.6×10^{3}	9.6×10^{4}	1.2×10^{4}
	V	Positive	1.7×10^{5}	1.8×10^{5}	3.3×10^{5}	1.9×10^{5}	1.6×10^{3}	9.1×10^{4}	6.7×10^{3}
